# Analysis of Volatile Organic Compounds in Milk during Heat Treatment Based on E-Nose, E-Tongue and HS-SPME-GC-MS

**DOI:** 10.3390/foods12051071

**Published:** 2023-03-02

**Authors:** Ning Yuan, Xuelu Chi, Qiaoyan Ye, Huimin Liu, Nan Zheng

**Affiliations:** 1 Key Laboratory of Quality & Safety Control for Milk and Dairy Products of Ministry of Agriculture and Rural Affairs, Institute of Animal Sciences, Chinese Academy of Agricultural Sciences, Beijing 100193, China; 2 State Key Laboratory of Animal Nutrition, Institute of Animal Sciences, Chinese Academy of Agricultural Sciences, Beijing 100193, China

**Keywords:** milk, VOCs, E-nose, E-tongue, HS-SPME-GC-MS

## Abstract

Volatile organic compounds (VOCs) make up milk flavor and are essential attributes for consumers to evaluate milk quality. In order to investigate the influence of heat treatment on the VOCs of milk, electronic nose (E-nose), electronic tongue (E-tongue) and headspace solid-phase microextraction (HS-SPME)–gas chromatography–mass spectrometry (GC-MS) technology were used to evaluate the changes in VOCs in milk during 65 °C heat treatment and 135 °C heat treatment. The E-nose revealed differences in the overall flavor of milk, and the overall flavor performance of milk after heat treatment at 65 °C for 30 min is similar to that of raw milk, which can maximize the preservation of the original taste of milk. However, both were significantly different to the 135 °C-treated milk. The E-tongue results showed that the different processing techniques significantly affected taste presentation. In terms of taste performance, the sweetness of raw milk was more prominent, the saltiness of milk treated at 65 °C was more prominent, and the bitterness of milk treated at 135 °C was more prominent. The results of HS-SPME-GC-MS showed that a total of 43 VOCs were identified in the three types of milk—5 aldehydes, 8 alcohols, 4 ketones, 3 esters, 13 acids, 8 hydrocarbons, 1 nitrogenous compound, and 1 phenol. The amount of acid compounds was dramatically reduced as the heat treatment temperature rose, while ketones, esters, and hydrocarbons were encouraged to accumulate instead. Furfural, 2-heptanone, 2-undecanone, 2-furanmethanol, pentanoic acid ethyl ester, 5-octanolide, and 4,7-dimethyl-undecane can be used as the characteristic VOCs of milk treated at 135 °C. Our study provides new evidence for differences in VOCs produced during milk processing and insights into quality control during milk production.

## 1. Introduction

Milk and milk products have a wide range of consumer groups in the world, to provide a large number of nutrients for human beings [1,2]. However, the European Food Safety Authority does not recommend directly consuming raw cow milk because it contains numerous pathogenic microorganisms [3,4]. Dairy quality regulators strongly recommend the processing of raw milk to improve food safety and increase milk’s shelf life [5]. Heat treatment is the most widely used processing technique in the dairy industry, and with increasing awareness of what constitutes “natural” or “unprocessed”, questions often arise about how heat treatment affects the nutritional value of milk and other dairy products [6]. Pasteurization at 65 °C for 30 min and ultra-high-temperature instantaneous sterilization at 135–150 °C for 2–8 s comprise the majority of raw milk processing techniques. The esters, alcohols, fatty acids, sulfur, carbonyls, and nitrogen compounds in raw milk are among the most important factors that influence consumers’ desire to purchase and play a significant role in the formation of milk flavor. The difference in some volatile substances depends on the processing technology, which can change the flavor of milk [7].

Temperature is an important factor affecting the volatile components of milk; during heat treatment, a series of physical and chemical changes occur that alter the flavor of milk [8]. For example, the main flavor compounds in raw milk, ethyl butanoate and ethyl hexanoate, disappear after pasteurization [9]. The content of 2-heptanone in pasteurized milk and UHT milk was much higher than that in high-temperature short-time sterilization milk [9]. According to research, the Maillard reaction, lipid degradation, and thermal denaturation of the whey protein and milk fat globule membrane were the primary contributors to the changes in milk flavor brought on by hot processing [10]. Thermal denaturation produces fatty acids, amino acids, and lactic acid, which are precursor substances to the formation of VOCs in milk [11]. The Maillard reaction was accelerated by the raw milk heating process, denaturing milk serum protein and increasing the content of sulfur compounds [12,13]. The lactose and amino groups in the protein are treated at high temperatures to produce sulfur-containing compounds that dissipate after a few weeks and are replaced by a characteristic “stale” odor [14,15]. The degradation of lipids in milk produces methyl ketones [2]. There was a strong association between methyl ketone concentration and heat damage indicators (furosine, lactulose, and undenatured whey protein) [16]. VOCs are an effective measure to assess milk quality changes, revealing chemical changes in the product well before the human perception threshold. Based on many studies, we can determine that heat treatment causes changes in milk flavor. However, there is a lack of combined analysis of the smell and taste of milk with heat treatment.

With the continuous development of science and technology, food flavor research by infrared spectroscopy, ultraviolet spectroscopy, gas chromatography–mass spectrometry, nuclear magnetic resonance spectroscopy, high-performance liquid chromatography–mass spectrometry, gas chromatography–ion mobility spectrometry, E-nose, E-tongue, metabolomics and other detection means is rich in the literature [17,18,19]. Among them, E-nose simulates the human sense of smell and can detect VOCs in samples non-destructively, while E-tongue simulates the human taste perception mechanism and can evaluate the overall taste of a sample. Some achievements have been made in the detection of broad VOCs, but they can not provide detailed information on VOCs [20,21]. The mature development of HS-SPME-GC-MS has allowed the technique to show significant advantages in analyzing VOCs in complex samples [22].

In this study, combined with the analytical advantages of E-nose, E-tongue and HS-SPME-GC-MS technology, taking raw milk as the control, the characteristics and differences in VOCs in milk at heat treatments of 65 °C for 30 min and 135 °C for 30 min provide theoretical support for the scientific utilization and processing of milk.

## 2. Materials and Methods

### 2.1. Sample Collection

In October 2022, milk samples were taken from seven healthy cows at a dairy farm in the Miyun district of Beijing, China. Sterile containers were used to transport all of the milk samples to the laboratory at 4 °C.

### 2.2. Heat Treatment

A volume of 400 mL of milk was taken and subjected to two different heat treatments. Treatment at 65 °C: Heat 200 mL milk in an aluminum pan in a 65 °C water bath for 30 min and cool to room temperature in ice cubes. Treatment at 135 °C: Heat 200 mL milk in an aluminum pan in a 135 °C oil bath for 30 min, then cool to room temperature in ice cubes. The thermal parameters of each treatment can be found in the Supplementary Materials (Tables S1 and S2).

### 2.3. E-Nose Analysis

VOCs in milk were identified using a PEN 3 E-nose (Airsense Analytics Inc., Schwerin, Germany). The performance description and the gas sensitivity range of the ten metal oxide sensors are shown in Table 1, adapted from Dong-Yu Shen [23].

The 8 mL milk sample was placed in a 20 mL headspace bottle and balanced in a constant-temperature water bath (Yuhua Instrument Co., Ltd., Zhengzhou, China) at 40 ± 2 °C for 5 min. The E-nose sampled 120 s at a flow rate of 300 mL/min. In order to ensure that the samples will not interfere with each other, the filtered air purification system will be used for 5 min after each sample injection. Each sample was repeated three times, and data with stable measurement results were selected for statistical analysis.

### 2.4. E-Tongue Analysis

The E-tongue detection was operated by Taste-Sensing System SA 402B (Intelligent Sensor Technology Co., Ltd., Atsugi, Japan). The taste perception of each sensor is shown in Table 2.

The accuracy of the test result will be affected if the milk contains too much fat during the E-tongue test. Therefore, it is necessary to dilute the milk and filter it with gauze after dilution. Take the diluted milk and place it in two 30 mL sample cups, and the sample in each cup will be measured four times. The tasteless threshold of each sensor of the E-tongue is based on the measured value of the reference solution as the standard output. The reference solution is composed of potassium chloride and tartaric acid. When the taste value of the sample is below the unscented point, the sample is said to have no taste.

### 2.5. Collection and Extraction of VOCs from Milk

Precisely 10 mL milk was added into the headspace bottle, and 1.00 g NaCl and 20 μL 2-methyl-3-heptanone (Steinheim, Germany) at 200 μg/L were added as the internal standard. After sealing the headspace bottle, balance it in a water bath at 40 °C for 30 min, stirring constantly. Milk VOCs were extracted using fiber coated with divinylbenzene/carboxen/polydimethylsiloxane (DVB/CAR/PDMS)-50/30 μm (Supelco, Bellefonte, PA, USA). After the headspace bottle was balanced for 30 min in a water bath at 40 °C, the extraction fiber was inserted into the bottle and left exposed to the upper half for 30 min for extraction. The fiber was extracted inserted into the GC-MS injection port, and splitless desorbed for five minutes at 250 °C.

### 2.6. GC-MS Analysis Conditions

Volatile compounds were detection using a gas chromatograph 7890A combined with a mass spectrometer 5975C (Agilent Technology, Santa Clara, CA, USA). The headspace extractions were then injected into the GC sample port equipped with a DB-WAX fused silica capillary column (30 m × 0.25 mm, 0.25 µm). GC conditions: Temperature program started from 30 °C for 6 min, increased at 100 °C/min to 240 °C, held for 5 min. The injection port temperature is 250 °C, the carrier gas was 99.99% pure helium with a flow rate of 1.0 mL/min. MS conditions: The electron ionization energy was 70 eV, and the ion source temperature was 230 °C. The transfer line temperature was set at 250 °C. A full scan mode was operated at a mass range of 45–450 amu with a quadrupole temperature of 150 °C.

### 2.7. Statistical Analysis

The qualitative analysis of VOCs was carried out using MS combined with the retention index. The NIST 14 database was used for MS to identify unknown compounds. The compound’s retention index (RI) was determined by measuring the retention time of C7-C40 n-alkanes. RI calculation is shown in Equation (1). The internal standard method was used for quantitative analysis of VOCs. One-way ANOVA (*p* < 0.05) was performed using SAS 9.2. Principal component analysis (PCA) and orthogonal partial least squares-discriminant analysis (OPLS-DA) were performed using SIMCA 14 software, PCA analysis was performed to select the principal components that could explain 80% of the total variance, and the input matrix was not automatically scaled. Origin 2018. https://www.omicstudio.cn/tool (accessed on 29 December 2022) was used for the Spearman correlation analysis.

(1) $$\mathrm{RI}=100Z+\frac{100\times \left[{\mathrm{TR}}_{x}{-\mathrm{TR}}_{Z}\right]}{{\mathrm{TR}}_{Z+1}{-\mathrm{TR}}_{z}}$$

where TR is the retention time, X is the compound to be analyzed, and Z and Z + 1 are the number of carbon atoms of the two n-alkanes before and after the compound to be analyzed, i.e., TR_Z_ < TR_X_ < TR_Z+1_.

## 3. Results

### 3.1. E-Nose Analysis

**Figure 1:**
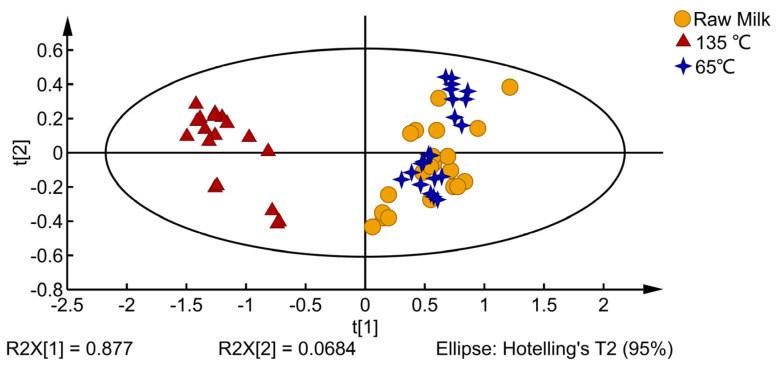
PCA of milk with different processing temperatures.

The electronic nose analysis results of milk at different processing temperatures are shown in Figure 1. PC1 and PC2 explained 94.54% of the total variance. Raw milk and the 65 °C-treated milk are on the positive axis of the PCA plot, and the 135 °C-treated milk is on the negative axis of the PCA plot. The milk samples of the raw milk and 65 °C-treated milk overlapped in the PCA diagram, and the overall flavor was similar, significantly different from that of the 135 °C-treated milk. From the E-nose data analysis, it can be inferred that temperature significantly affects the VOCs of milk.

**Figure 2:**
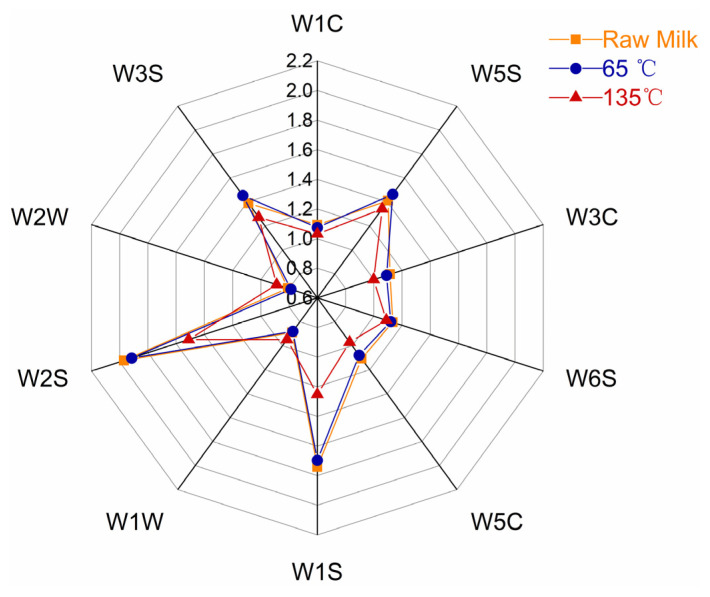
Radar map of VOCs in milk processed using different temperatures.

The E-nose has a distinct advantage in detecting overall changes in the sample compound. The intensity of the sensor response represents the intensity of the compound odor, with a high response intensity indicating a strong compound odor [24]. The E-nose radar diagram of milk VOCs at different processing temperatures is shown in Figure 2. The intensity of the reaction was not significantly different between milk samples treated at 65 °C and raw milk samples. After the raw milk is processed at 135 °C, the response intensity of the W1W and W2W sensors increased; however, the response intensity of the other eight sensors decreased, especially when the response of W1S and W2S sensors decreased dramatically. Sulfur compounds were detected by W1W and W2W sensors, while organic matter such as methyl and carbon–oxygen compounds were detected by W1S and W2S sensors, indicating that high-temperature heating promoted the synthesis of sulfur compounds in milk and reduced the content of organic matter.

### 3.2. E-Tongue Analysis

The results of using E-tongue are shown in Table 3. The sourness has a tasteless threshold of −13, saltiness has a tasteless threshold of −6, and other indicators have a tasteless threshold of 0. When the taste value of the sample is lower than the tasteless threshold, this indicates that the sample has no such taste; when it is higher than the tasteless threshold, this indicates that the sample has this taste [25]. All samples have higher response values for bitterness, umami and sweetness, but lower response values for Aftertaste-B and Aftertaste-A. The 135 °C-treated milk had a significantly higher bitterness response value than the raw milk and the 65 °C-treated milk (*p* < 0.05). The sweetness response differed—the 135 °C-treated milk was significantly less sweet than the raw milk and the 65 °C-treated milk (*p* < 0.05). This point was also confirmed in the subsequent GC-MS detection. The furfural content increased significantly due to the increase in processing temperature, and furfural presented a burnt bitterness to the outside. Milk becomes more bitter and less sweet as it is processed at higher temperatures.

**Figure 3:**
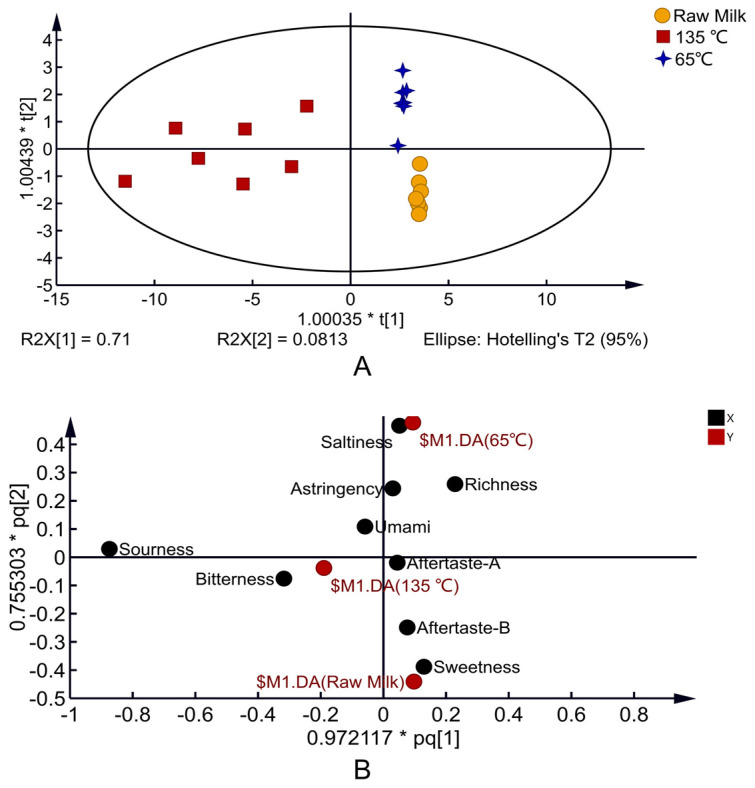
OPLS-DA 2D score map of milk E-tongue with different temperatures (**A**) and load diagram (**B**).

As shown in Figure 3A, the OPLS-DA model was established according to the test results of E-tongue, andthe model (R2X = 0.972, R2Y = 0.806, Q2 = 0.765) showed good discriminant and predictive ability. The E-tongue accurately distinguished milk samples with different heat treatments, which indicated that temperatures lead to apparent differences in milk taste. The load diagram explains the taste contribution of different heat-treated milk. Raw milk is located in the fourth quadrant, close to sweet and Aftertaste-B, indicating that sweet and Aftertaste-B are the primary taste manifestations of raw milk; 65 °C-treated milk is located in the first quadrant, close to saltiness, and its primary taste is saltiness; 135 °C-treated milk is located in the third quadrant, close to bitterness, and its primary taste is bitterness (Figure 3B).

### 3.3. HS-SPME-GC-MS Analysis

**Figure 4:**
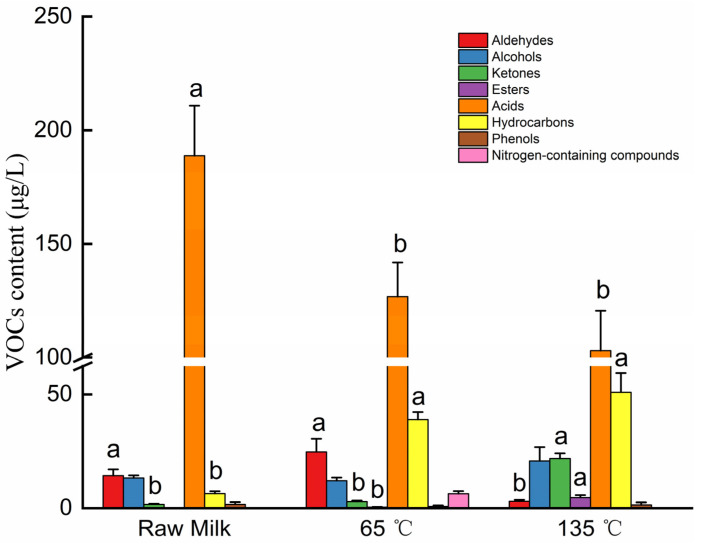
Changes in VOC content in milk under different temperatures. Different lowercase letters represent significant differences at *p* < 0.05.

After siloxane compounds were removed due to the loss of extraction fibers and columns, GC-MS identified 43 VOCs, as shown in Table 4, which included 5 aldehydes, 8 alcohols, 4 ketones, 3 esters, 13 acids, 8 hydrocarbons, 1 nitrogenous compound, and 1 phenol. As shown in Figure 4, the content of acid compounds in raw milk was significantly higher than in the 65 °C-treated milk and the 135 °C-treated milk (*p* < 0.05). In the 135 °C-treated milk, the amounts of ketones, esters, and hydrocarbons significantly increased, while the amounts of aldehydes significantly decreased (*p* < 0.05). The sensory properties of milk are affected by the significant variation in VOC content caused by the difference in heat treatment.

**Figure 5:**
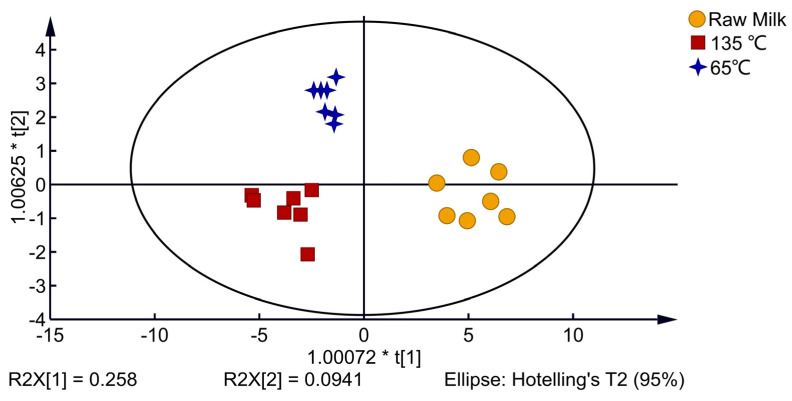
OPLS-DA 2D score map of milk with different processing temperatures.

As shown in Figure 5, the OPLS-DA model was used for discriminant analysis of VOCs produced by milk with different processing temperatures. All three of the model’s quality parametersR2X = 0.843, R2Y = 0.839, and Q2 = 0.858are greater than 0.5, indicating that the model is capable of making accurate predictions and discriminations. The raw milk samples are between quadrants one and four of the 2D score chart, the 65 °C-treated milk samples are in quadrant two and the 135 °C-treated milk samples are in quadrant three, with the different processing methods of dairy samples clearly separated into different areas. The results indicated that VOCs in milk at different heat treatment temperatures were significantly different.

**Figure 6:**
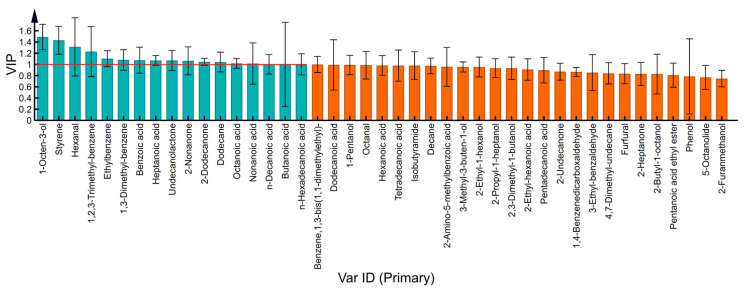
VIP chart of milk VOCs at different processing temperatures.

As shown in Figure 6, through the OPLS-DA model, 17 VOCs with VIP > 1 were screened. These were 17 VOCs—1 aldehyde (hexanal), 1 alcohol (1-octen-3-ol), 2 ketones (2-nonanone and 2-dodecanone), 1 ester (undecanolactone), 7 acids (butanoic acid, heptanoic acid, octanoic acid, nonanoic acid, n-decanoic acid, benzoic acid, and n-hexadecanoic acid) and 5 hydrocarbons (ethylbenzene, 1,3-dimethyl-benzene, dodecane, styrene, and 1,2,3-trimethyl-benzene).

### 3.4. Correlation Analysis between Thermal Parameters and VOCs

**Figure 7:**
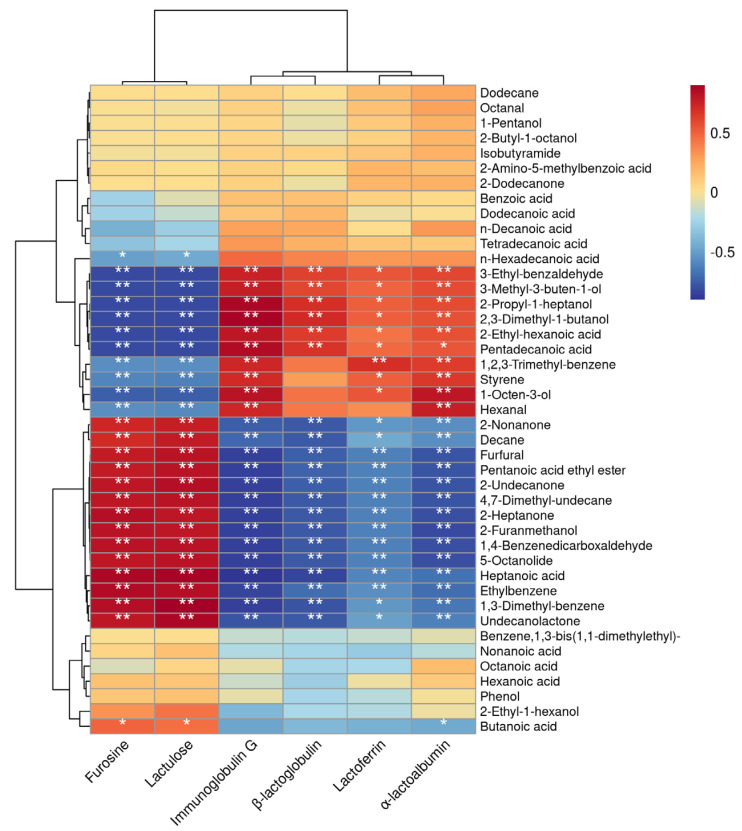
Correlation analysis between thermal parameters and VOCs (** *p* < 0.01, * *p* < 0.05).

The correlation analysis between the thermal sensitivity index and VOCs was shown in Figure 7. The compounds 3-ethyl-benzaldehyde, 3-methyl-3-buten-1-ol, 2-propyl-1-heptanol, 2,3-dimethyl-1-butanol, and 2-ethyl-hexanoic acid showed highly significant negative correlations with the heat-sensitive indicators furosine and lactulose when the heat treatment temperature of milk increased (*p* < 0.01); a highly significant positive correlation with immunoglobulin G, α-lactoalbumin, and β-lactoglobulin (*p* < 0.01); and a significant positive correlation with lactoferrin (*p* < 0.05). The compounds furfural, pentanoic acid ethyl ester, 2-undecanone, 4,7-dimethyl-undecane, 2-heptanone, 2-furanmethanol, 1,4-benzenedicarboxaldehyde, 5-octanolide, heptanoic acid, and ethylbenzene showed highly significant positive correlations with the heat-sensitive indicators furosine and lactulose as the heat treatment temperature of milk increased (*p* < 0.01), and a highly significant negative correlation with immunoglobulin G, α-lactoalbumin, β-lactoglobulin, and lactoferrin (*p* < 0.01).

## 4. Discussion

This study aimed to explore the effects of different heat treatment on milk VOCs and to achieve this goal through E-nose, E-tongue, and SPME-GC-MS. Milk is rich in nutrients such as fat, protein, and lactose, which are broken down into fatty acids, amino acids, etc., after different processing methods, and these are the precursor substances of VOCs which determine the flavor of milk [11]. The degree to which milk is heated affects the formation of furfurals [26]. In this study, furfural was found in the 135 °C-treated milk at a concentration of 2.55 ± 0.59 μg/L (Table 4), but not in the 65 °C-treated milk and raw milk. Many studies have gradually confirmed that furfural is a product of milk after heating, and a recent study showed that an amount of 15 mg/kg furfural in pasteurized milk treated at 70 °C for 15 s [27]. Furfural itself exhibits a burnt, roasted taste. In this study, the formation of furfural also causes the bitterness in milk to become the main taste expression in 135 °C-treated milk. At the same time, studies have shown that bitterness is related to the hydrolysis of whey protein. YGLF, IPAVF, LLF, and YPFPGPIPN are amino acid sequences of bitter peptides, and a high degree of hydrolysis often has increases bitterness intensity [28,29,30]. Jo’s research found that skim milk with a higher serum protein content had stronger sulfur and eggy flavor and higher sulfur compounds [31]. Lee’s research shows that ultra-high-temperature instantaneously sterilized milk may have a longer shelf life than pasteurized milk, but it produces a more sulfurous and eggy taste, which may be unacceptable to consumers [32]. The present study also confirmed this theory that furfural, immunoglobulin G, β-lactoglobulin, lactoferrin, and α-lactoalbumin showed a highly significant negative correlation (Figure 7). The milk’s bitterness is more pronounced at higher levels of protein hydrolysis.

After heat treatment, lipids will produce a large number of methyl ketones, which are also products of milk treated at high temperatures. Additionally, milk heated at 135 °C for 5 s undergoes a more thorough Maillard reaction with a higher concentration of Maillard products and methyl ketones than milk heated at 75 °C for 15 s [15]. Jo showed that differences in flavor formation caused by the Maillard reaction and protein denaturation during hot processing affected the flavor quality of super pasteurized milk and resulted in significant flavor differences between direct steam injection and indirect heating of milk [2]. In this study, the maximum amount of methyl ketones was found in 135 °C-treated milk, with an amount of 13.75 ± 1.79 μg/L, 5.72 ± 0.75 μg/L, and 2.43 ± 0.42 μg/L 2-heptanone, 2-nonanone, and 2-undecanone, respectively. Although the methyl ketone compounds can reflect the heating degree of milk to some extent, the content of methyl ketone compounds, such as 2-heptanone, will decrease with the extension of milk storage time after high-temperature treatment [33]. Methyl ketones have been shown to have a positive correlation with the amount of harmful lactosyl lysine molecules in heat-treated milk, making it an effective method for determining the quality of milk [16]. Acid compounds comprise a large proportion of milk, with 13 VOCs, and are important in forming milk odor. The increase in milk processing temperature leads to a gradual decrease in the total amount of acid compounds (Figure 4). The content of lactone compounds is also among the important factors affecting milk flavor, and milk flavor is generally negatively correlated with the content of lactone [34]. In the different treatments of this study, no lactones were detected in raw milk; however, two lactones, 5-octanolide and undecanolactone, were detected in 135 °C-treated milk, which further explained the flavor deterioration of 135 °C-treated milk. The correlation analysis revealed that 5-octanolide and undecanolactone showed a significant positive correlation with furosine and lactulose and a significant negative correlation with protein parameters. The detection 5-octanolide and undecanolactone also indicates a reduction in milk quality.

Many studies have proved that thermal processing significantly affects the VOC composition of milk, which is related to the Maillard reaction [35]. Triglycerides account for approximately 98% of milk’s fatty acids, but triglycerides are not volatile and heat processing converts them into volatile compounds [36]. Short- and medium-chain fatty acids in milk butterfat produce many aroma compounds. Aldehydes, acids, and alcohols are converted from unsaturated fatty acids, while esters are converted from free fatty acids [37]. Fat content has an impact on the VOC richness of milk. In UHT milk, the types of VOCs of low-fat milk are lower than those of high-fat milk, and acetoin, 2-nonanone, and 2-heptanone were positively linear correlated with fat content [36]. Therefore, the fat content in milk has a significant impact on milk flavor, and the control of milk flavor is primarily a matter of fat regulation. From a broad perspective, milk flavor is influenced by many things, such as the cow diet, breed, natural enzymes in milk, microorganisms and processing conditions that may alter milk flavor balance [38,39]. Zhou found that different microbial starter cultures had a significant effect on the flavor of yogurt, and the content of benzaldehyde and acetoin could be significantly increased by using L. helveticus H9 strain [40]. The Sun study showed that the levels of acetaldehyde, 2,3-butanedione, acetoin, butyric acid, decanoic acid, hexanoic acid, and octanoic acid were significantly higher at 37 °C than at 42 °C, indicating that low temperatures contribute to the formation of flavor compounds [41].

High-temperature treatment of milk to inactivate harmful bacteria is an effective means used worldwide. However, it also promotes the transformation of nutrients such as fat and protein in milk and changes the flavor of milk. The OPLS-DA model screened out 17 compounds with metabolic differences due to temperature changes (Figure 6), and high-temperature treatment intensified the accumulation of furfural (baked odor), methyl ketones (fruity, fresh sweet, and spicy odor), esters (sweet fruity, fatty and creamy odor), and hydrocarbons, leading to the change in milk flavor.

## 5. Conclusions

This study analyzed the flavor compounds in milk processed using different heat treatment by E-nose, E-tongue and HS-SPME-GC-MS technology. The results show that the overall flavor performance of milk after heat treatment at 65 °C for 30 min is similar to that of raw milk, which can maximize the preservation of the original taste of milk. The milk’s flavor changed significantly as a result of the heat treatment, with the sweetness being more prominent in the raw milk, saltiness in the 65 °C-treated milk, and bitterness in the 135 °C-treated milk. HS-SPME-GC-MS identified 43 VOCs in raw, 65 °C-, and 135 °C-treated milk—5 aldehydes, 8 alcohols, 4 ketones, 3 esters, 13 acids, 8 hydrocarbons, 1 nitrogen compound, and 1 phenol. Furfural, 2-heptanone, 2-undecanone, 2-furanmethanol, pentanoic acid ethyl ester, 5-octanolide, and 4,7-dimethyl-undecane are unique compounds in 135 °C-treated milk, reflecting the characteristics of VOCs from milk that has been treated at high temperatures for a long time. We are thinking about how to improve milk nutrition and extend shelf life while ensuring food safety, and we will continue our in-depth research in areas such as processing and packaging materials.

## Figures and Tables

**Table 1:** E-nose sensor description [23].

No.	Designation	Performance Description	Main Sensitive Substance	Detection Threshold (mL/m^3^)
1	W5S	nitrogen oxides	NO_2_	1
2	W1S	methyl	CH_4_	100
3	W2S	alcohols, ketones and aldehydes	CO	100
4	W3C	ammonia	C_6_H_6_	10
5	W1C	benzene	C_7_H_8_	10
6	W5C	short-chain aromatic compounds and olefin	C_3_H_8_	1
7	W1W	sulfur compounds	H_2_S	1
8	W2W	organic sulfides	H_2_S	1
9	W6S	hydrogen	H_2_	100
10	W3S	long-chain alkanes	CH_4_	100

**Table 2:** E-tongue sensor performance description.

NO	Designation	Performance
1	CT0	Saltiness
2	CA0	Sourness
3	AAE	Umami, Aftertaste-U
4	AE1	Astringency, Aftertaste-A
5	C00	Bitterness, Aftertaste-B
6	GL1	Sweetness

**Table 3:** Taste value of milk E-tongue with different processing temperatures.

	Sourness	Saltiness	Bitterness	Astringency	Aftertaste-B	Aftertaste-A	Umami	Sweetness	Aftertaste-U
Tasteless	−13	−6	0	0	0	0	0	0	0
Raw milk	−45.71 ± 0.14 c	0.18 ± 0.44 b	7.81 ± 0.19 b	1.94 ± 0.18 b	1.00 ± 0.07 a	0.37 ± 0.03 a	6.79 ± 0.11 b	11.08 ± 0.23 a	4.91 ± 0.11 b
65 °C	−41.41 ± 0.15 b	3.29 ± 0.44 a	7.88 ± 0.25 b	3.19 ± 0.14 a	0.18 ± 0.19 b	0.33 ± 0.02 a	7.21 ± 0.08 ab	8.92 ± 0.17 b	6.59 ± 0.22 a
135 °C	5.77 ± 4.36 a	1.43 ± 1.70 ab	15.10 ± 2.10 a	2.37 ± 0.71 ab	0.07 ± 0.08 b	0.18 ± 0.04 b	7.73 ± 0.95 a	8.35 ± 0.35 c	1.18 ± 0.26 c

Different lowercase letters represent significant differences at *p* < 0.05.

**Table 4:** The types and contents of VOCs in milk under different processing temperatures.

	NO	Compound	Retention Time	CAS#	Formula	RI_(Ref)_	RI	Raw Milk (μg/L)	65 °C (μg/L)	135 °C (μg/L)
Aldehydes	1	Hexanal	8.386	66-25-1	C_6_H_12_O	1095	1008	13.57 ± 2.57	22.28 ± 5.34	ND
	2	Octanal	12.677	124-13-0	C_8_H_16_O	1289	1272	ND	2.53 ± 0.42	ND
	3	Furfural	15.205	98-01-1	C_5_H_4_O_2_	1439	1452	ND	ND	2.55 ± 0.59
	4	3-Ethyl-benzaldehyde	18.318	34246-54-3	C_9_H_10_O		1700	0.89 ± 0.25	ND	ND
	5	1,4-Benzenedicarboxaldehyde	22.701	623-27-8	C_8_H_6_O_2_		2119	ND	ND	0.47 ± 0.09
Alcohols	1	2-Butyl-1-octano	4.719	3913-02-8	C_12_H_26_O	1853		ND	3.90 ± 0.89	ND
	2	2,3-Dimethyl-1-butanol	4.722	19550-30-2	C_6_H_14_O			0.63 ± 0.12	ND	ND
	3	2-Propyl-1-heptanol	6.221	10042-59-8	C_10_H_22_O		862	5.54 ± 0.90	ND	ND
	4	3-Methyl-3-buten-1-ol	12.103	763-32-6	C_5_H_10_O	1236	1234	2.21 ± 0.24	ND	ND
	5	1-Pentanol	12.191	71-41-0	C_5_H_12_O	1258	1240	ND	1.96 ± 0.24	ND
	6	1-Octen-3-ol	15.124	3391-86-4	C_8_H_16_O	1458	1446	2.39 ± 0.47	1.73 ± 0.24	ND
	7	2-Ethyl-1-hexanol	15.665	104-76-7	C_8_H_18_O	1484	1487	2.62 ± 0.62	4.59 ± 0.36	4.25 ± 1.05
	8	2-Furanmethanol	17.694	98-00-0	C_5_H_6_O_2_	1664	1649	ND	ND	16.58 ± 6.22
Ketones	1	2-Heptanone	10.81	110-43-0	C_7_H_14_O	1184	1152	ND	ND	13.75 ± 1.79
	2	2-Nonanone	14.277	821-55-6	C_9_H_18_O	1389	1383	1.75 ± 0.28 b	2.45 ± 0.43 b	5.72 ± 0.75 a
	3	2-Undecanone	17.025	112-12-9	C_11_H_22_O	1599	1594	ND	ND	2.43 ± 0.42
	4	2-Dodecanone	17.062	6175-49-1	C_12_H_24_O		1597	ND	0.47 ± 0.03	ND
Esters	1	Pentanoic acid ethyl ester	20.915	539-82-2	C_7_H_14_O_2_		1939	ND	ND	2.82 ± 0.70
	2	5-Octanolide	21.141	698-76-0	C_8_H_14_O_2_	1956	1961	ND	ND	0.95 ± 0.42
	3	Undecanolactone	23.369	710-04-3	C_11_H_20_O_2_		2190	ND	0.52 ± 0.07 b	0.94 ± 0.16 a
Acids	1	Butanoic acid	17.309	107-92-6	C_4_H_8_O_2_	1637	1616	4.49 ± 0.47 b	4.63 ± 0.62 b	10.57 ± 2.61 a
	2	2-Amino-5-methylbenzoic acid	18.811	2941-78-8	C_8_H_9_NO_2_		1744	ND	40.43 ± 9.89	ND
	3	Hexanoic acid	19.79	142-62-1	C_6_H_12_O_2_	1851	1832	5.49 ± 0.91	6.90 ± 1.09	6.65 ± 1.81
	4	2-Ethyl-hexanoic acid	20.901	149-57-5	C_8_H_16_O_2_	1962.5	1938	1.05 ± 0.22	ND	ND
	5	Heptanoic acid	20.908	111-14-8	C_7_H_14_O_2_	1960	1938	ND	0.75 ± 0.21	1.91 ± 0.24
	6	Styrene	21.988	124-07-2	C_8_H_16_O_2_	2050	2045	8.63 ± 1.28	7.56 ± 1.56	8.88 ± 1.36
	7	Nonanoic acid	23.011	112-05-0	C_9_H_18_O_2_	2174	2151	1.02 ± 0.25	0.63 ± 0.17	1.24 ± 0.31
	8	n-Decanoic acid	23.984	334-48-5	C_10_H_20_O_2_	2279	2257	10.74 ± 1.65	6.39 ± 1.13	7.03 ± 2.33
	9	Benzoic acid	25.419	65-85-0	C_7_H_6_O_2_	2433	2421	5.03 ± 0.68 a	2.25 ± 0.53 b	4.20 ± 1.13 ab
	10	Dodecanoic acid	25.817	143-07-7	C_12_H_24_O_2_	2502	2469	7.83 ± 1.38	4.42 ± 0.82	7.66 ± 2.75
	11	Tetradecanoic acid	27.539	544-63-8	C_14_H_28_O_2_	2692	2679	41.21 ± 6.08 a	24.56 ± 5.45 b	28.67 ± 8.32 ab
	12	Pentadecanoic acid	28.528	1002-84-2	C_15_H_30_O_2_	2803	2785	54.86 ± 11.44	ND	ND
	13	n-Hexadecanoic acid	29.75	57-10-3	C_16_H_32_O_2_	2876	2894	48.43 ± 6.50 a	28.23 ± 4.29 b	26.20 ± 4.32 b
Hydrocarbons	1	Decane	6.225	124-18-5	C_10_H_22_		863	ND	8.24 ± 1.31	8.93 ± 1.48
	2	4,7-Dimethyl-undecane	7.369	17301-32-5	C_13_H_28_		946	ND	ND	7.23 ± 1.76
	3	Ethylbenzene	9.263	100-41-4	C_8_H_10_	1122	1059	ND	7.27 ± 0.74 b	14.87 ± 2.82 a
	4	1,3-Dimethyl-benzene	9.587	108-38-3	C_8_H_10_	1140	1078	ND	7.94 ± 1.68 b	17.05 ± 4.01 a
	5	Dodecane	10.995	112-40-3	C_12_H_26_		1163	ND	9.01 ± 1.01	ND
	6	Styrene	12.001	100-42-5	C_8_H_8_	1254	1227	1.59 ± 0.43	2.31 ± 0.47	ND
	7	1,2,3-Trimethyl-benzene	12.39	526-73-8	C_9_H_12_	1267	1253	1.70 ± 1.21	1.26 ± 0.23	ND
	8	Benzene,1,3-bis(1,1-dimethylethyl)-	14.783	1014-60-4 90	C_14_H_22_		1420	3.22 ± 0.27	3.05 ± 0.32	3.00 ± 0.28
Nitrogen-containing compounds	1	Isobutyramide	8.94	563-83-7	C_4_H_9_NO		1009	ND	6.43 ± 1.07	ND
Phenols	1	Phenol	21.421	108-95-2	C_6_H_6_O	2008	1988	1.75 ± 0.91	0.86 ± 0.40	1.55 ± 1.00

“ND” indicates not detected. Different lowercase letters represent significant differences at *p* < 0.05. RI_(Ref)_: documented retention index. RI: retention index.

## Data Availability

Data is contained within the article or Supplementary Materials.

## Supplementary Materials

The following supporting information can be downloaded at: https://www.mdpi.com/article/10.3390/foods12051071/s1, Table S1. Method for the determination of thermal parameters; Table S2. Thermal parameters for each treatment group. References [42,43,44,45,46] are cited in the supplementary materials.
