# Supplementary material for: Analysis of Volatile Organic Compounds in Milk during Heat Treatment Based on E-Nose, E-Tongue and HS-SPME-GC-MS
Source: Foods. 2023 Mar 2;12(5):1071. doi: 10.3390/foods12051071 (PMC10001307; doi:10.3390/foods12051071)
Supplement: Supplementary file 1 [file foods-12-01071-s001.zip › foods-2221615-supplementary.pdf]

Supplementary materal

Table S1. Method for the determination of thermal parameters.

Thermal parameters	Determination method	Number
Furosine<br>Lactulose	Identification of reconstituted milk in pasteurized and UHT milk	NY/T 939—2016 [42]
Lactoferrin	Determination of lactoferrin in milk and dairy products-Liquid chromatography method	T/TDSTIA 006—2019 [43]
α-lactoalbumin	Determination of α-lactoalbumin in milk and dairy products-Liquid chromatography method	T/TDSTIA 002—2021 [44]
β-lactoglobulin	Determination of β-lactoglobulin in milk and dairy products-Liquid chromatography method	T/TDSTIA 007—2019 [45]
Immunoglobulin G	Determination of immunoglobulin G in milk and dairy products-High performance Liquid chromatography method	T/TDSTIA003-2021 [46]

Table S2. Thermal parameters for each treatment group.

	Furosine (mg/100g protein)	Lactulose (mg/kg)	Lactoferrin (mg/kg)	α-lactoalbumin (mg/kg)	β-lactoglobulin (mg/kg)	Immunoglobulin G (mg/kg)
Raw Milk			160.21	1241.43	3745.16	266.20
65 °C	15.98	19.38	54.53	1140.45	3140.60	133.17
135 °C	239.07	685.49		100.92	123.00	
